# Antecedent infections in Fisher syndrome: sources of variation in clinical characteristics

**DOI:** 10.1007/s00415-019-09308-x

**Published:** 2019-04-06

**Authors:** Michiaki Koga, Masahiko Kishi, Toshihiro Fukusako, Naomi Ikuta, Masayuki Kato, Takashi Kanda

**Affiliations:** 1 Department of Neurology and Clinical Neuroscience, Yamaguchi University Graduate School of Medicine, Minami-kogushi 1-1-1, Ube, Yamaguchi 755-8505 Japan; 2 Neurology, Internal Medicine, Sakura Medical Center, Toho University, Chiba, Japan; 3 Yachiyo Rehabilitation Hospital, Chiba, Japan; 4 Department of Neurology, Yamaguchi Prefectural Grand Medical Center, Yamaguchi, Japan; 5 Department of Neurology, Ube-Kosan Central Hospital, Yamaguchi, Japan; 6 Department of Neurology, Shimonoseki Medical Center, Yamaguchi, Japan

**Keywords:** Fisher syndrome, Antecedent infection, *Haemophilus influenzae*, *Campylobacter jejuni*, Cytomegalovirus, Intercellular adhesion molecule 1

## Abstract

The clinical features of Guillain–Barré syndrome (GBS) are highly variable, according to the type of antecedent infection. Although a major GBS phenotype, Fisher syndrome (FS), has been shown to be preceded by infections similar to those preceding GBS, whether or not the clinical features in FS also vary according to antecedent infection remains unclarified. Frequent antecedent infections among this study of 70 FS patients included *Haemophilus influenzae* [*n* = 15 (21%)], *Campylobacter jejuni* [*n* = 10 (14%)], and cytomegalovirus (CMV) [*n* = 6 (8.6%)]. Compared with other FS patients, *H. influenzae*-seropositive FS patients more frequently had a history of prior upper respiratory tract infection; double vision as the initial symptom; and, except for oculomotor disturbance, more rarely showed cranial nerve involvement. *C. jejuni*-related FS occurred predominantly in younger male patients and characteristically presented with blurred vision. According to GBS disability scale, CMV-related FS tended to be more severe, although every patient received immunotherapy. Serum anti-GQ1b IgG antibodies were detected in most cases, regardless of antecedent infection type. At the nadir of illness, the most frequent diagnosis in *H. influenzae*-related cases was “pure FS” without limb weakness or central nervous system involvement (71%), in *C. jejuni*-related cases “incomplete FS” such as acute ophthalmoparesis with or without ataxia (60%), and in CMV-related cases (50%) advanced conditions such as GBS overlap and Bickerstaff brainstem encephalitis. These findings indicate that the type of preceding infection determined the neurological features of FS. CMV-related FS appeared to be similar to *H. influenzae*- and *C. jejuni*-related FS regarding anti-GQ1b antibody-mediated pathogenesis, as opposed to CMV-related GBS.

## Introduction

Guillain–Barré syndrome (GBS) is a postinfectious autoimmune-mediated neuropathy, the clinical manifestations of which greatly vary among individual cases [1]. An at least partial spectrum of antecedent infections has been determined, which indicates that *Campylobacter jejuni* is the most frequent causal agent of GBS [2]. To clarify the pathogenesis of GBS, classifying GBS into subgroups based on the type of antecedent infection, such as “*C. jejuni*-associated GBS” and “cytomegalovirus (CMV)-associated GBS” could be important, because antecedent infections are closely associated with neurological phenotypes and specific autoantibodies [3]. For example, GBS patients with CMV infection showed higher blood levels of soluble intercellular adhesion molecule 1 (sICAM-1) than the levels in other GBS patients, which suggests that a cytotoxic mechanism is involved in the development of CMV-associated GBS [4].

Fisher syndrome (FS) is a major clinical phenotype of GBS. It is characterized neurologically by the triad of ophthalmoplegia, ataxia, and areflexia and immunologically by the frequent presence of anti-GQ1b IgG autoantibodies [5]. Our previous case-controlled serological study showed that *C. jejuni* and *Haemophilus influenzae* were the major identified agents of antecedent infection in FS [6]. However, unlike for GBS, close associations of clinical features with types of antecedent infections are yet to be clarified for FS, perhaps because of stereotypical consideration with regard to a relatively uniform clinical picture and presence of specific autoantibodies (anti-GQ1b antibodies), and also of limitations of our previous study which did not include cases of incomplete forms of FS, such as acute ophthalmoparesis without ataxia, and of advanced FS that included Bickerstaff brainstem encephalitis (BBE) [6]. The aim of this study was to clarify whether or not the clinical and laboratory features of FS vary as they do in GBS, according to antecedent infections.

## Methods

### Study participants

This study consisted of a cohort of 70 patients with FS, all of whom had undergone a medical examination or were referred to Yamaguchi University Hospital for assessment of the presence of serum anti-glycolipid antibodies between 2001 and 2017. The clinical criteria for enrollment have been reported previously [7]. However, unlike our previous studies [6, 7], this study included patients with FS in the broad sense to recruit a wide range of patients with FS-related conditions as follows: without ataxia or areflexia or both (namely “incomplete FS”) and those who initially presented with the triad of manifestations characteristic of FS and then developed generalized muscle weakness (namely, FS/GBS overlap) or disturbance in consciousness (final diagnosis being BBE) or both. To collect clinical data from cases not our own, we requested information on other relevant patients from their primary physicians, which included neurological findings and GBS disability scale (GDS) [8] at the nadir of illness. For the analysis of sICAM-1 level, serum samples from 31 patients with GBS were also used in addition to those from FS patients. The study protocol was approved by the Institutional Ethics Committee at Yamaguchi University Hospital.

### Serological analysis

Serum IgM and IgG antibodies against isolated gangliosides (GM2, GM1, GD1a, GalNAc-GD1a, GD1b, GT1a, GT1b, or GQ1b; 10 pmol/well) and ganglioside complexes (GM1/GD1a and GM1/GT1a) were measured by enzyme-linked immunosorbent assay (ELISA) as described elsewhere [9]. IgG subclasses of anti-GQ1b and anti-GT1a antibodies were examined as reported previously [10]. Antecedent *C. jejuni* and *H. influenzae* infections were investigated by an in-house ELISA method, as reported elsewhere [6]. CMV, Epstein–Barr virus, and *Mycoplasma pneumoniae* infections were serologically identified using commercially available kits, as described elsewhere [6]. Serum level of sICAM-1 was measured using a commercially available ELISA kit (R&D Systems, Inc., Minneapolis, USA).

### Data analysis

Differences in frequencies between groups were analyzed using the Fisher exact test. Differences in medians were examined by the Mann–Whitney *U* test. Differences were considered significant for *P *< 0.05 in two-sided tests. Statistical calculations were performed by SPSS 19 software (IBM Japan, Tokyo, Japan).

## Results

### Serology and symptoms of antecedent infections

Among 70 patients with FS, serological evidence of recent infection with *H. influenzae, C. jejuni*, CMV, and *M. pneumoniae* was seen in 15 (21%), 10 (14%), 6 (8.6%), and 1 (1.4%) patient, respectively. One patient showed seropositivity for both *H. influenzae* and CMV. No agent of antecedent infection was identified in 39 (56%) patients. Patients with *H. influenzae* or *C. jejuni* infections were younger [median age, 43 year (*P *= 0.06) and 41 year (*P *= 0.02), respectively] than the other patients. The male–female ratio of patients with *C. jejuni* infections was disproportionally male (8:2) compared to the gender ratio of the other patients (27:33, *P *= 0.04) (Table 1).

**Table 1:** Neurological findings at admission

	Total	Antecedent infectious agents			
		*H. influenzae*	*C. jejuni*	CMV	None identified
Number	70	15^a^	10	6^a^	39
Age, median (range)	55 (2–76)	43 (2–69)	41 (12–58)*	49 (23–65)	60 (9–76)**
Gender, male/female	35/35	7/8	8/2***	2/4	19/20
Admission, days after onset^a^	4 (1–28)	3 (1–11)	4 (2–11)	3 (1–11)	4 (1–28)
Neurological findings at admission^b^					
Abducens-dominancy in OP	32/52 (62%)	9/13 (69%)	4/7 (57%)	3/4 (75%)	17/29 (59%)
Internal OP	35/69 (51%)	7 (47%)	7 (70%)	5 (83%)	16/39 (41%)
Facial palsy	14/66 (21%)	0/13****	3 (30%)	1 (17%)	9/38 (24%)
Bulbar palsy	15/68 (22%)	1/13 (7.7%)	2 (20%)	3 (50%)	8 (21%)
Cranial nerve palsy except for OP	22/66 (33%)	1/13 (7.7%)*****	4 (67%)	3 (50%)	13/37 (35%)
Neck weakness	6/59 (10%)	0/13	2/8 (25%)	1 (17%)	3/33 (9.1%)
Limb weakness	7 (10%)	0	1 (10%)	1 (17%)	4 (10%)
Ataxia	58/69 (84%)	14 (93%)	6 (60%)	6 (100%)	33/38 (87%)
Sensory disturbance	22/65 (34%)	5/14 (36%)	3 (30%)	4 (67%)	9/35 (26%)
Autonomic disturbance	4/60 (6.7%)	1/12 (8.3%)	0/8	0	3/34 (8.8%)
GBS disability scale, median (range)	2 (1–5)	2 (1–4)	2 (1–4)	3 (2–4)	2 (1–4)

*CMV* cytomegalovirus, *OP* opthalmoplegia, *GBS* Guillain–Barré syndrome

*Lower than others (*P *= 0.02)

**Higher than others (*P *= 0.0004)

***Larger proportion of males than others (*P *= 0.04)

****Rarer than others (*P *= 0.03)

*****Rarer than others (*P *= 0.03)

^a^Including one patient who was double positive for *H. influenzae* and CMV

^b^First visit to neurology department in patients who had not been hospitalized

Any symptom of infection preceded the onset of neurological manifestations in 66 (94%) of 70 patients. Respiratory tract symptoms were the most frequent in patients with *H. influenzae* infection [15 of 15 (100%)], which was a significantly higher proportion of patients than the proportion of other FS patients with respiratory tract symptoms (62%, *P *= 0.002). In contrast, gastrointestinal symptoms were the most frequent in patients with *C. jejuni* infection [8 of 10 (80%)], which was a significantly higher proportion of patients than the proportion of other FS patients with gastrointestinal symptoms (20%, *P *= 0.0004). Among the six patients with CMV seropositivity, four (67%) had a history of prior respiratory tract symptoms, one (17%) had gastrointestinal symptoms, and one (17%) did not have any preceding symptom of infection.

### Initial symptoms and neurological findings at admission

The most frequent initial neurological symptom was double vision, regardless of antecedent infection, but this manifestation was pronounced in patients with *H. influenzae* (87%) compared to patients with *C. jejuni* (40%) and CMV (50%) infections. It is noteworthy that three (30%) of ten patients with *C. jejuni* infections, in whom obvious mydriasis was observed at admission, initially presented with blurred vision without double vision or ptosis. Neurological findings at admission (or first visit to the neurology department by patients who had not been hospitalized) are shown in Table 1. Lack of cranial nerve palsy except for ophthalmoplegia was characteristic for patients with *H. influenzae* infection. Frequent occurrence of sensory disturbance was characteristic for patients with CMV infection, although the difference was not statistically significant (compared to the others: *P *= 0.09). The differences between GDS of the patients with each type of antecedent infection at admission were not remarkable.

### Immunotherapy, final diagnosis, and disability scores at nadir (Table 2)

As immunotherapy, intravenous immunoglobulin therapy was administered to most patients. The median times from the onset to the nadir of illness for patients with *H. influenzae* or *C. jejuni* infections were both 6 days, whereas it tended to be longer for those with CMV infection (9 days). The neurological findings at the nadir varied, depending on the type of antecedent infection: patients with *H. influenzae* infection presented with the FS triad only, and 50% of patients with CMV infection had bulbar palsy. Variation in neurological disturbances was well reflected by the final diagnoses: “pure FS” (FS triad without limb weakness or disturbance of the central nervous system disturbance) in 71% of patients with *H. influenzae* infection, “incomplete FS” (acute ophthalmoparesis without ataxia and/or a-/hyporeflexia) in 60% of *C. jejuni* patients, and FS/GBS overlap or BBE in 50% of CMV patients.

**Table 2:** Immunotherapies and clinical features at nadir

	Total	Antecedent infectious agents			
		*H. influenzae*	*C. jejuni*	CMV	None identified
Number	70	15	10	6	39
Immunotherapies					
Intravenous immunoglobulin	52/69 (75%)	10/14 (71%)	8 (80%)	6 (100%)	28 (72%)
Steroid pulse	2/69 (2.9%)	0/14	0	0	2 (5.1%)
Duration from onset to nadir, median (range)	6 (2–30)	6 (3–11)	6 (3–11)	9 (3–14)	8 (2–30)
> 7 days	29/68 (43%)	2/13 (15%)*	2 (20%)	4 (67%)	20 (51%)
Neurological findings at nadir					
Conscious disturbance	2/66 (3.0%)	0/14	1/9 (11%)	1 (17%)	0/38
Bulbar palsy	15/66 (23%)	1/14 (7.1%)	3/9 (33%)	3 (50%)	8/38 (21%)
Limb weakness	13/66 (20%)	2/14 (14%)	1/9 (11%)	2 (33%)	8/38 (21%)
Artificially ventilated	2/67 (3.0%)	0/14	0/9	0	1/38 (2.6%)
Diagnosis at nadir					
“Pure FS”^a^	39/69 (57%)	10/14 (71%)	2 (20%)**	3 (50%)	25 (64%)
“Incomplete FS” (AO ± ataxia)	12/69 (17%)	2/14 (14%)	6 (60%)***	0	4 (10%)
FS/GBS overlap or BBE	18/69 (26%)	2/14 (14%)	2 (20%)	3 (50%)	10 (26%)
GDS at nadir, median (range)	3 (1–5)	3 (1–4)	3 (1–4)	4 (3–4)	2 (1–5)
GDS ≧ 3	36/67 (54%)	7/13 (54%)	5 (50%)	6 (100%)****	18/38 (47%)
Duration of hospitalization, median (range)	20 (2–57)	15 (5–36)	17 (6–48)	24 (15–36)	20 (2–57)
> 2 weeks	40/64 (63%)	7/14 (50%)	6/9 (67%)	6 (100%)	21/35 (60%)

*CMV* cytomegalovirus, *FS* Fisher syndrome, *AO* acute ophthalmoparesis, *GBS* Guillain–Barré syndrome, *BBE* Bickerstaff brainstem encephalitis, *GDS* GBS disability scale

*Rarer than others (*P *= 0.02)

**Rarer than others (*P *= 0.01)

***More frequent than others (*P *= 0.001)

****More frequent than others (*P *= 0.02)

^a^Having the triad of ophthalmoplegia, ataxia, and a/hyporeflexia without limb weakness and central nervous system disturbance

Regarding GDS and duration of hospitalization, the patients with *H. influenzae* and *C. jejuni* infections were similar (median GDS, both 3; median hospitalization duration, 15 and 17 days, respectively). These data were in contrast with those with CMV infection, in whom GDS tended to be higher (4 in all cases) and the duration of hospitalization longer (median 24 days: more than 2 weeks in all). Patients without an identified antecedent infection were positioned between the patients with *H. influenzae*/*C. jejuni* infections and the patients with CMV infection with respect to time from onset to nadir of illness, neurological findings at the nadir, and duration of hospitalization.

### Laboratory data

IgG antibodies against GQ1b and GT1a were detected in most cases, regardless of antecedent infection, and the pattern of other anti-ganglioside IgG antibodies did not considerably differ between the groups (Table 3). However, the IgG subclasses of anti-GQ1b antibodies were unevenly distributed, as follows: patients with *H. influenzae* and *C. jejuni* infections most often showed IgG1 anti-GQ1b seropositivity, whereas those with CMV infection only showed IgG3 anti-GQ1b seropositivity. Anti-GM2 IgM antibodies were negative in all patients. Differences in the results of cerebrospinal fluid analysis between the groups were not significant.

**Table 3:** Laboratory data

	Total	Antecedent infectious agents			
		*H. influenzae*	*C. jejuni*	CMV	None identified
Number	70	15	10	6	39
IgG Abs against					
GQ1b	65 (93%)	14 (93%)	10 (100%)	5 (83%)	36 (92%)
GT1a	67 (96%)	15 (100%)	10 (100%)	5 (83%)	37 (95%)
GM1	5 (7.1%)	0	2 (20%)	1 (17%)	2 (5.1%)
GD1a	5 (7.1%)	0	2 (20%)	0	3 (7.7%)
GD1b	14 (20%)	3 (20%)	1 (10%)	2 (33%)	7 (18%)
GM1/GT1a complex	67 (96%)	15 (100%)	10 (100%)	5 (83%)	37 (95%)
GM1/GD1a complex	9 (13%)	1 (6.7%)	2 (20%)	2 (33%)	3 (7.7%)
IgG subclass of anti-GQ1b Abs^a^					
Only IgG1	29/65 (45%)	8 (53%)	6/9 (67%)	2 (33%)	14/36 (39%)
Only IgG3	27/65 (42%)	4 (27%)	3/9 (33%)	4 (67%)	16/36 (44%)
Both IgG1 + IgG3	9/65 (14%)	3 (20%)	0/9	0	6/36 (17%)
Cerebrospinal fluid					
Increased protein conc	23/54 (43%)	4 (27%)	3/7 (43%)	2/5 (40%)	13/27 (48%)
Increased cell count	5/54 (9.3%)	2 (13%)	0/7	0/5	2/27 (7.4%)

*CMV* cytomegalovirus, *Abs* antibodies, *conc* concentration

^a^Anti-GT1a antibodies in cases of negative anti-GQ1b antibodies

**Fig. 1:**
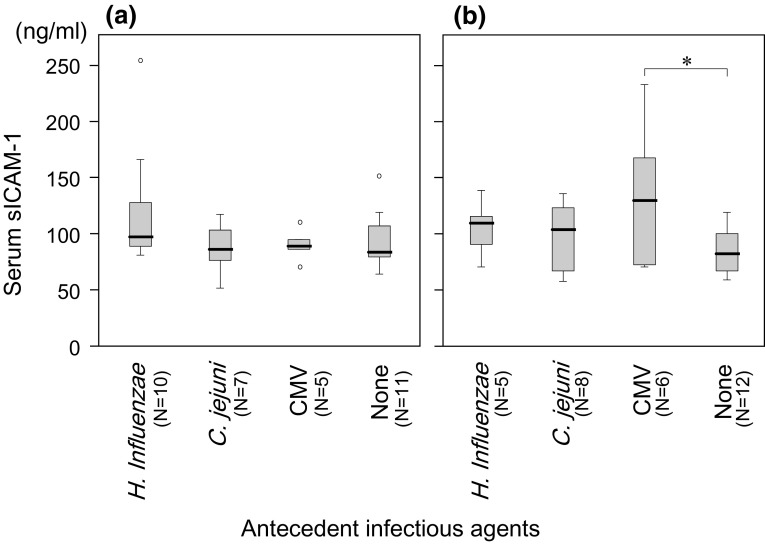
Serum concentration of soluble intercellular adhesion molecule 1 (sICAM-1) in **a** Fisher syndrome (FS) and **b** Guillain–Barré syndrome (GBS). The differences in serum concentrations between FS patients depending on antecedent infectious agents were not significant (**a**), whereas the GBS patients who had serological evidence of antecedent cytomegalovirus (CMV) infection showed a significantly higher concentration of sICAM-1 than patients with GBS in whom antecedent infectious agents had not been identified (*P = 0.04) (**b**). [None] denotes the patients in whom antecedent infectious agents had not been identified by the serological assays described in the text

As in the previous study [4], the serum concentration of sICAM-1 tended to be higher in patients with CMV-associated GBS than those with other antecedent infection-associated GBS (Fig. 1). In contrast, no apparent increase of sICAM-1 concentration was seen in CMV-associated FS.

## Discussion

Because of the frequent detection of anti-GQ1b antibodies, FS tends to be considered as a relatively uniform clinical entity, and there have been few studies regarding variability in the clinical picture of FS. This study has clearly shown that FS has a markedly variable clinical picture, and that three antecedent infections (*H. influenzae*, *C. jejuni*, and CMV) are closely associated not only with antecedent symptoms of infection, but also with neurological features and disability scores, as follows: (1) *H. influenzae*-related FS occurred after upper respiratory tract symptoms, patients presented with double vision as an initial symptom, showed “pure FS” at the nadir of illness without cranial nerve involvement other than oculomotor disturbance and limb weakness, and showed mild disability according to the GDS; (2) *C. jejuni*-related FS occurred predominantly in younger male patients after gastrointestinal symptoms, remained “incomplete FS” with manifestations such as acute ophthalmoparesis with mild disability, and sometimes was characterized by an exceptional initial symptom of blurred vision; and (3) CMV-related FS tended to manifest bulbar palsy and sensory disturbance, and to advance to FS/GBS overlap or BBE, with severe disability at the nadir of illness despite immunotherapy.

A large cohort study that included 194 patients with anti-GQ1b IgG antibodies showed that various phenotypes, including acute ophthalmoparesis, FS, BBE, and their manifestations overlapping with GBS, formed a distinct disease entity, namely “anti-GQ1b IgG antibody syndrome” [11]. However, that study did not clarify why such clinical variation occurs, except for limb weakness, which was partially associated with common existence of anti-GM1 or anti-GD1a antibodies in addition to anti-GQ1b antibodies. To the best of our knowledge, our study is the first to identify an antecedent infection as a causative factor in determining the subtypes of “IgG anti-GQ1b antibody syndrome”.

It remains unclear why neurological variation occurs in FS despite the common occurrence of anti-GQ1b IgG antibodies. A possible explanation might be the cross-reactivity of anti-GQ1b antibodies with ganglioside complexes. Depending on antibody specificities, anti-GQ1b antibodies are divided into three types: GQ1b-specific, GM1/GQ1b-reactive, and GD1a/GQ1b-reactive [12], and such diverse specificities might lead to clinical differences between FS patients [13]. In our simple analysis that only assessed two anti-ganglioside-complex antibodies (anti-GM1/GT1a and anti-GM1/GD1a), we failed to find differences in antibody specificities according to the antecedent infection. Additional detailed analyses of a large number of anti-ganglioside complex antibodies are needed to evaluate the theory that diverse antibody specificities might lead to clinical differences between FS patients.

Among the three identified subtypes of FS, *H. influenzae*- and *C. jejuni*-related FS showed several similar characteristics, including onset of illness in younger patients, short period of time between onset to the nadir of illness (median 6 days), neurological deficits limited to the triad of FS with mild disability at the nadir, and IgG1 subclass predominance of anti-GQ1b antibodies. In contrast, CMV-related FS manifested different characteristics, including longer period of time between onset to nadir of illness (median 9 days); neurological deficits beyond the triad of FS, including sensory disturbance and limb weakness; increased severity in disability at the nadir of illness, and IgG3 subclass predominance of anti-GQ1b antibodies. These clinical features of CMV-related FS are similar to those of CMV-related GBS, which was characterized by longer period of time from onset to nadir of illness (median 10 days) compared to *C. jejuni*-related GBS (median 7 days), frequent cranial nerve palsy and sensory disturbance, and severe disability [14–16]. Although anti-GM2 IgM antibodies and anti-moesin IgG antibodies have been proposed to be the autoantibodies associated with CMV-related GBS [17–19], findings casting doubt on their contributory roles in the pathogenesis of GBS have also been reported [20, 21], and therefore a role for pathogenic autoantibodies remains unclear in CMV-related GBS. This issue, as well as the finding of elevated concentrations of serum sICAM-1 in patients with CMV-related GBS [4], indicate the possible role of a cytotoxic mechanism rather than a humoral mechanism in the development of CMV-related GBS. Therefore, we had at first hypothesized that anti-GQ1b IgG antibodies would not be present in CMV-related FS. However, this study found that anti-GQ1b antibodies were as frequent in patients with CMV-related FS as they were in patients with FS preceded by other infections, and that serum sICAM-1 concentration was not elevated in patients with CMV-related FS in contrast to patients with CVM-related GBS. We concluded that a humoral mechanism could be a causative factor in CMV-related FS as well.

We previously reported that the presence of IgG1 subclass of antibodies against GM1 and other motor gangliosides was closely associated with poor recovery in GBS patients [10, 22]. In the current study, information on disability scale during recovery phase was not available, and therefore it remains unclear whether IgG1 subclass of anti-GQ1b antibodies is also associated with poor recovery in FS. We instead compared the duration of hospitalization between the groups and found that patients with IgG1 anti-GQ1b antibodies tended to need rather shorter hospitalization [median 17 (5–55) days] than those without IgG1 antibodies [20 (8–57) days: *P *= 0.11]. This suggests that IgG1 subclass of anti-GQ1b antibodies is not an indicator for poor recovery in FS, but further investigation should be necessary regarding this matter.

This study has limitations. First, this study included a relatively small number of FS patients (*N* = 70), only six patients with CMV-related FS were analyzed, and statistical significance could not be confirmed for several situations. Second, inaccurate clinical data and selection bias cannot be completely denied, because the data were derived from a multicenter cohort. To reduce the possible problems, we contacted the involved primary physicians, and without divulging information on the serology on antecedent infections, asked them to answer a questionnaire as well as to provide a summary on their patients. Finally, no antecedent infectious agent was identified in more than a half of FS patients in our study, and a wider range of possible antecedent infections should be investigated. Two case–control studies from Bangladesh and The Netherlands reported a significant association for hepatitis E virus (HEV) infection with GBS [23, 24]. One FS patient positive for anti-HEV IgM antibodies has also been identified among six Japanese FS patients [25]; however, our preliminary study did not find a patient with serological evidence of recent HEV infection among 65 Japanese patients with FS (Koga, unpublished data).

In conclusion, our data strongly suggest that the type of preceding infection determines the neurological features of FS. In clinical practice, cases of CMV-FS especially should be actively treated, because they tended to progress to severe disability. Moreover, CMV-related FS appeared to be similar to *H. influenzae*- and *C. jejuni*-related FS regarding anti-GQ1b antibody-mediated pathogenesis as opposed to CMV-related GBS.
